# Plant bZIPs in Root Environmental Adaptation: From Single-Cell Expression Atlas to Functional Insights

**DOI:** 10.3390/ijms27020568

**Published:** 2026-01-06

**Authors:** Menglan Xu, Linping Zhang, Jingyan Wang, Shuxin Gan, Yan Xiong, Yanlin Liu, Zhenzhen Zhang

**Affiliations:** Fujian Provincial Key Laboratory of Haixia Applied Plant Systems Biology, Haixia Institute of Science and Technology, College of Life Sciences, Fujian Agriculture and Forestry University, Fuzhou 350002, China; 15673123482@163.com (M.X.); 18058302106@163.com (L.Z.); jingyanwang0313@163.com (J.W.); ganshuxin45@163.com (S.G.); yanxiong@fafu.edu.cn (Y.X.)

**Keywords:** bZIP transcription factors, scRNA sequencing, bZIP regulatory modules, root growth and adaptation, environmental signals

## Abstract

Plant roots interact dynamically with complex environments, and their capacity to adapt is crucial for growth, development, survival, and productivity. Basic leucine zipper (bZIP) transcription factors have emerged as key regulators in managing the root’s response to various environmental signals. The shift from bulk tissue analysis to single-cell RNA sequencing (scRNA-seq) has enabled the creation of a highly detailed expression atlas for root bZIPs, significantly enhancing our understanding of their functions. This review first summarizes the classification and structural features of bZIPs in *Arabidopsis*, and compares representative members with their orthologs in cereal crops. Next, we integrate the expression patterns of various *bZIP* members in root cells and clarify their roles through single-cell expression profiling. Furthermore, we delineate characterized bZIP regulatory modules that respond to signals spanning light, hormones, nutrients, and stresses, thereby orchestrating transcriptional reprogramming to facilitate plant adaptation. By combining single-cell omics with functional genetics, we reveal how bZIPs control critical processes, including responses to light signals, hormonal interactions, nutrient uptake and balance, and reactions to abiotic stresses. Ultimately, this integrated perspective highlights the potential for targeting bZIP transcription factors in the development of climate-resilient crops with optimized root systems, thereby enabling them to adapt to changing environmental conditions.

## 1. Introduction

The root system serves as a crucial interface between plants and their environment, acting as a vital sensor and responder to dynamic and challenging soil conditions [1]. The capacity of roots to adaptively develop in response to both abiotic (nonliving) and biotic (living) stresses is a key factor influencing plant fitness, agricultural resilience, and productivity [2]. A primary goal in plant biology is to understand the molecular mechanisms that enable plants to adapt remarkably to their environment. At the core of these regulatory mechanisms are transcription factors, with the basic leucine zipper (bZIP) family being one of the most extensive and versatile groups of transcriptional regulators in higher plants [3]. bZIP proteins are characterized by a basic region for DNA binding and an adjacent leucine zipper domain that allows for dimerization. These proteins function as master integrators of environmental and hormonal signals, binding to specific cis-elements to direct complex transcriptional programs that control plant stress responses and development [3,4].

Traditionally, our understanding of bZIP functions in roots has been largely derived from bulk-tissue analyses. While these conventional approaches have provided valuable insights, they inevitably mask the rich cellular diversity inherent to the root system [5]. This limitation is particularly relevant given that different root cell layers—including the epidermis, cortex, endodermis, and stele—perform specialized functions in nutrient uptake, stress perception, and developmental patterning. The recent advent of single-cell RNA sequencing (scRNA-seq), however, has transformed our ability to dissect this complexity. By offering an unprecedented, high-resolution “atlas” of gene expression at the cellular level, scRNA-seq enables the identification of transcription factor functions with remarkable spatial precision [5]. A compelling example of this power comes from maize, where the integration of transcriptome-wide association studies with scRNA-seq revealed ZmbZIP89—a bZIP transcription factor specifically expressed in cortical and epidermal cells—as a key regulator of lateral root elongation and drought tolerance [5]. Thus, through the lens of single-cell technologies, we are now transitioning from a generalized organ-level perspective toward a finely resolved, cell-type-specific understanding of bZIP functions—ushering in a new era of precision in plant functional genomics.

Recent studies have significantly advanced our understanding of how bZIP transcription factors mediate root-environment interactions through diverse molecular mechanisms. One prominent example is bZIP29, which regulates root development by modulating cell wall properties and influencing cell proliferation in the root meristem [6]. Beyond developmental control, bZIP factors play equally important roles in nutrient sensing and metabolic regulation. bZIP11 exemplifies this function through its unique sucrose-sensing mechanism, where ribosome stalling at its upstream open reading frame (uORF2) enables the plant to monitor energy status [7]. Furthermore, bZIP11 integrates low-energy signals with auxin-mediated pathways to control primary root growth [7,8]. Metabolomic analyses further confirm that bZIP11 and related transcription factors coordinate nutrient allocation during root tip development [7]. The regulatory complexity of bZIP networks is further enhanced by sophisticated post-translational mechanisms. Under drought stress, for instance, the ABA signaling pathway activates the phosphorylation of AREB1 (bZIP36) and AREB2 (bZIP38), thereby enhancing plant drought resistance [9,10,11]. These findings illustrate how bZIP transcription factors form multi-layered regulatory networks that integrate developmental cues, nutrient availability, and environmental stresses to coordinate root growth and adaptation precisely.

This review systematically synthesizes the rapidly advancing field of bZIP transcription factors in plant root biology. First, we will explore how emerging single-cell expression atlases are revolutionizing our understanding of the spatial and temporal dynamics of *bZIP* expression across diverse root cell types. Subsequently, we will integrate these spatial insights with mechanistic studies that elucidate how specific bZIP modules—often regulated by post-translational modifications and complex dimerization networks—enable roots to adapt to environmental challenges, including drought, salinity, and nutrient deficiency.

By strategically connecting cutting-edge single-cell omics with functional genetics and physiological evidence, this review aims to establish a comprehensive framework for understanding how bZIP transcription factors coordinately orchestrate root adaptation. Ultimately, this synthesis review will highlight the significant potential of bZIP factors as promising targets for developing climate-resilient crops.

## 2. The Plant bZIP Transcription Factor Family: Classification and Protein Structural Characteristics

### 2.1. A Systematic Classification of Plant bZIPs

**Figure 1:**
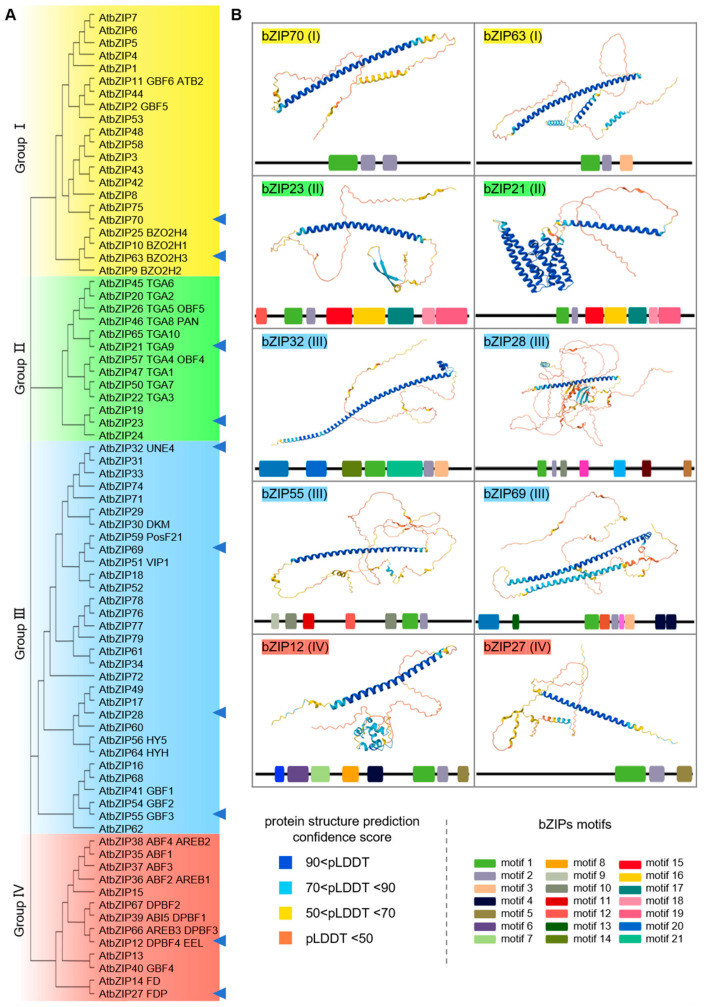
Phylogenetic analysis and structural features of bZIP transcription factors in *Arabidopsis*. (**A**) A total of 78 bZIP transcription factors are present in the *Arabidopsis* genome. They can be classified into four groups (Groups I–IV) based on conserved motifs, sequence homology, and evolutionary relationships. (**B**) Three-dimensional structures of the representative bZIP proteins from each group were displayed. The protein structures were predicted by AlphaFold 3. Colors in the structural models indicate prediction confidence scores, while schematic blocks represent the composition and arrangement of motifs on the secondary structures. Information on other bZIP proteins is provided in Supplementary Figure S1.

bZIP transcription factors are widely involved in regulating growth and development, metabolism, and responses to various biotic and abiotic stresses in plants [3]. Due to its important functions, the bZIP family of proteins has been extensively studied in plants. Following the completion of sequencing several plant genomes, bZIPs are typically categorized through systematic identification and evolutionary analysis across the genome [12,13]. bZIP proteins are typically classified based on conserved motifs within the bZIP domain, sequence homology, and phylogenetic relationships. Following a similar approach, we constructed a phylogenetic tree to analyze protein structural conservation among these sequences, which led to their classification into four distinct groups in *Arabidopsis,* consistent with the groupings reported by Droge-Laser et al. [3]. Using the AlphaFold three online tool, we selected 2–4 representative proteins from each group to predict their three-dimensional structures (Figure 1 and Figure S1). For example, bZIP70 and bZIP63, representatives of Group I, share conserved motifs and exhibit relatively simple three-dimensional structures. These primarily consist of a single long alpha helix and irregular coils (Figure 1 and Figure S1). Additionally, bZIP63 features several smaller alpha-helices segments interspersed within its intrinsically disordered regions. Group II proteins, represented by bZIP23 and bZIP21, also share similar conserved motifs. Most Group II members are characterized by multiple long alpha helices arranged successively (Figure 1 and Figure S1), as seen in bZIP21. However, a subset of Group II, consisting of bZIP19, bZIP23, and bZIP24, displays protein structures more like those in Group I, with the key difference being the presence of several beta sheets in their irregular coiled regions (Figure 1 and Figure S1). Group III proteins, including bZIP32, bZIP28, bZIP55, and bZIP69, constitute the largest group within the bZIP family. Although they exhibit significant differences in their motifs, their protein structures show a degree of similarity (Figure 1 and Figure S1). Like Group I, their structures feature more complex and diverse irregular coils (Figure 1 and Figure S1). Group IV bZIP proteins, such as bZIP12 and bZIP27, not only possess a single long alpha helix but also often include multiple smaller alpha helices arranged in succession within the irregular coil regions (Figure 1 and Figure S1). This analysis shows that the four *Arabidopsis* bZIP groups exhibit distinct structural features. These differences align with similarities in their domain features, suggesting they may function redundantly (within same groups) and diversely (among groups) in regulating plant growth and environmental adaptation.

This classification framework, based on model plant species, provides valuable references for studying bZIP family proteins in other plant species. Subsequent studies on a variety of herbaceous and woody plants, including *Platycodon grandiflorum* [14], rice [15], alfalfa [16], maize [17], *Phoebe bournei* [12] and poplar [18], found that although the number of genes varies by species specificity, most of their *bZIP* genes can be classified into the four groups defined in *Arabidopsis*. This means the classification scheme is universally applicable across plants.

**Figure 2:**
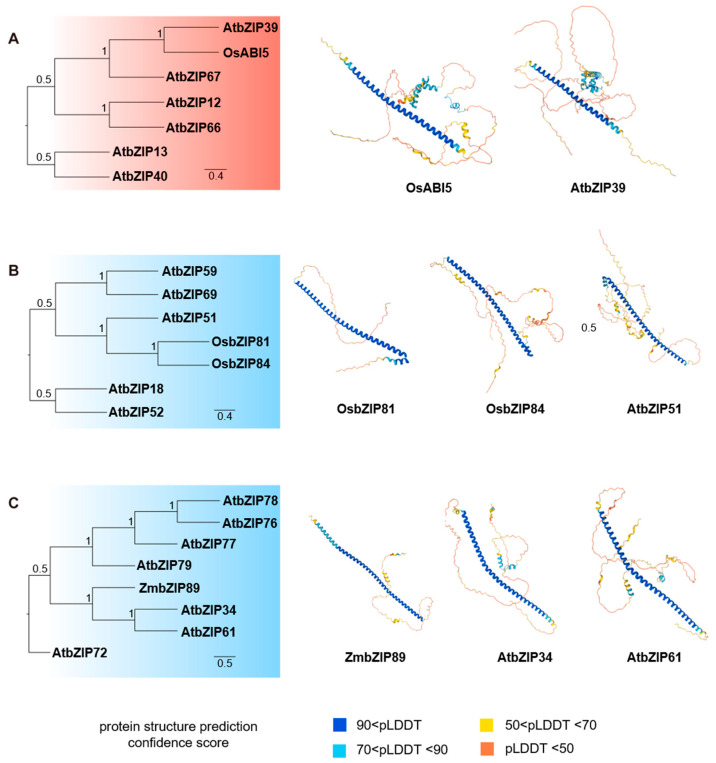
Phylogenetic analysis and structural features of representative bZIP transcription factors in maize, rice and *Arabidopsis*. (**A**) OsABI5 and its *Arabidopsis* orthologs. (**B**) OsbZIP81/84 and their *Arabidopsis* orthologs. (**C**) ZmbZIP89 and its orthologs in *Arabidopsis*. The protein structures were predicted by AlphaFold 3. Colors in the structural models indicate prediction confidence scores.

To investigate the evolutionary conservation of bZIP transcription factors in plants, we compared the protein structures of well-characterized bZIPs from crops with their *Arabidopsis* counterparts. Phylogenetic analysis and predicted protein structures revealed strong structural conservation of bZIP domains across monocots and dicots. For instance, OsABI5 is closely related to *Arabidopsis* bZIP39, whereas OsbZIP81 and OsbZIP84 show high structural similarity to *Arabidopsis* bZIP51; similarly, ZmbZIP89 aligns with *Arabidopsis* bZIP34 and bZIP61 (Figure 2). These conserved relationships suggest that these proteins may possess comparable DNA-binding specificities and dimerization preferences. Indeed, the core functional regions—the basic DNA-binding domain and the leucine zipper dimerization motif—are notably conserved, as reflected in their close clustering within phylogenetic subfamilies.

Despite overall structural conservation, sequence variation outside the core bZIP domain likely contributes to functional divergence, enabling species-specific adaptations in stress responses, development, and tissue-specific expression. This structural preservation across *Arabidopsis* and major crops underscores the translational relevance of *Arabidopsis* as a model system. Research on *Arabidopsis* bZIPs not only elucidates fundamental regulatory mechanisms but also establishes a predictive framework for engineering bZIP networks in agronomically important species, ultimately supporting efforts to improve stress tolerance and crop yield.

### 2.2. Structural Architecture of Plant bZIP Transcription Factors

The main feature of the bZIP protein is its bZIP domain, which consists of approximately 60 amino acids. This domain is divided into two closely related functional regions: the basic region and the leucine zipper region [3]. The basic region typically comprises approximately 16 amino acids and is rich in lysine and arginine in its N-terminal part, which carry positive charges. This characteristic is essential for its electrostatic attraction to the negatively charged DNA backbone. Importantly, there is a conserved sequence, the “DNA contact motif” (N-X_7_-R/K-X_9_), within this region. In this motif, the asparagine and arginine or lysine residues directly bind to the bases in the large groove of DNA by forming a specific hydrogen bond. This enables the bZIP protein to recognize specific cis-acting elements that contain the core sequence ACGT [19].

The leucine zipper region is an excellent design for protein–protein interactions. It consists of a series of heptad repeats, and typically, a hydrophobic leucine (or other hydrophobic amino acids, such as valine or isoleucine) is located at the seventh position of each repeat unit [19]. When two α-helices like that are arranged in parallel, these periodic leucine residues form stable coiled helical dimers by hydrophobic forces, like the zipper. This dimerization ability is the cornerstone of the functional diversity of bZIP proteins [20]. Proteins can form homodimers or heterodimers with members of the same or different groups [20]. Different dimer combinations may significantly alter binding affinity and specificity for DNA, thereby greatly expanding the regulatory potential of the bZIP family [21]. Specifically, different dimer combinations prefer to recognize distinct ACGT variants [22]. The bZIPs that recognize the G-box (CACGTG) [23] usually form homodimers. Recognition of C-box (GACGTC) or A-box (TACGTA) often requires a specific heterodimer combination [22]. This “combinatorial regulation” mechanism enables the construction of complex and precise gene regulatory networks using a limited number of transcription factors through dimerization, thereby adapting to ever-changing internal and external environments.

## 3. A Single-Cell Atlas of bZIP Expression in the Root: Spatial Patterning and Environmental Responses

Although bZIP proteins within the same group share structural features and partially conserved functions, individual members exhibit distinct functional specialization. To systematically investigate this specificity, we combined single-cell transcriptomic data from the *Arabidopsis* root cell atlas (https://rootcellatlas.org, accessed 11 September 2025) [24,25,26,27,28,29,30] with bulk RNA-seq datasets (https://bar.utoronto.ca/eplant/, accessed 28 September 2025) [31,32,33] capturing responses to environmental signals. Our integrated approach revealed that *bZIP* genes display characteristic expression patterns across root developmental zones—meristem, elongation, and mature regions—while simultaneously showing dynamic regulation under various environmental conditions. Based on these observations, we propose that the functional roles of *bZIP* genes in root development and adaptation can be inferred from their spatial expression specificity and environmental responsiveness. Specifically, we hypothesize that *bZIP* members that exhibit both high spatial specificity and strong environmental induction are key regulators orchestrating root adaptive responses, implicating them in distinct developmental processes and stress adaptation mechanisms.

### 3.1. bZIP Genes in Group I

**Figure 3:**
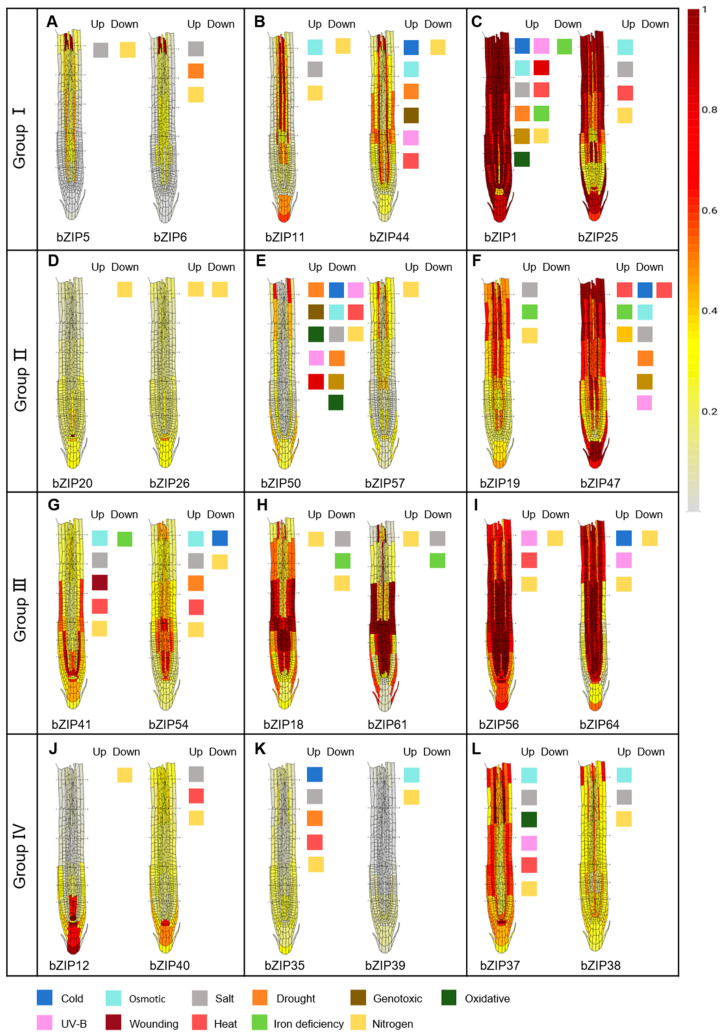
Single-cell expression atlas and environmental regulation of bZIP transcription factors in the *Arabidopsis* root tip. Spatial expression patterns of representative *bZIP* genes from four subfamilies (Groups I–IV) are shown, with genes clustered according to expression profile similarity within each group. Corresponding environmental signals that regulate these genes are identified, with color coding indicating transcriptional responses (both induction and repression) to specific stimuli across different root cell layers. Information about other bZIP proteins can be found in Supplementary Figure S2.

Group I *bZIP* transcription factors exhibit spatially distinct expression profiles that align with their specialized roles in root development and stress adaptation. For example, *bZIP5*, *bZIP6*, *bZIP7*, and *bZIP9* are predominantly expressed in vascular tissues (pericycle, xylem, phloem) of the mature zone and respond to nitrogen and salt stress (Figure 3A–C and Figure S2A), suggesting their involvement in nutrient transport and abiotic stress responses. A second expression pattern is observed in meristematic regions, where *bZIP11*, *bZIP44*, and *bZIP63* show elevated expression around the quiescent center (QC) and root cap. bZIP44 additionally localizes to epidermal and cortical tissues, while *bZIP63* marks endodermal and stem cell niche regions. Their regulation by salt stress and nitrogen signals (Figure 3A–C and Figure S2A) indicates roles in coordinating cell differentiation with environmental conditions. In contrast, *bZIP1*, *bZIP2*, and *bZIP25* exhibit broad expression across multiple root zones and cell types, coupled with responsiveness to diverse stresses including osmotic, salt, heat, and nitrogen signals (Figure 3A–C and Figure S2A). This widespread expression pattern suggests these factors function as central regulators integrating multiple physiological processes—from cell growth and differentiation to stress adaptation—throughout the root system.

### 3.2. bZIP Genes in Group II

Moving to Group II, *bZIP* genes display a spectrum of localization patterns that reflect division of labor across root zones. Three members—*bZIP20*, *bZIP26*, and *bZIP46*—are predominantly expressed in quiescent center cells and are regulated by nitrogen signaling (Figure 3D–F and Figure S2B), suggesting their involvement in maintaining stem cell homeostasis under varying nutrient conditions. In contrast, another subset, including *bZIP65*, *bZIP21*, *bZIP57*, *bZIP50*, and *bZIP22*, shows preferential expression in differentiated tissues of the elongation and mature zones (epidermis, cortex, and endodermis) (Figure 3D–F and Figure S2B). These genes respond to salt stress, iron deficiency (Figure 3D–F and Figure S2B), and nitrogen signals, indicating roles in environmental adaptation within mature root tissues. Notably, *bZIP47* and *bZIP19* display broad expression across all root zones—from the root cap to mature tissues—and are regulated by both iron deficiency and nitrogen signaling (Figure 3D–F and Figure S2B). Their widespread expression pattern suggests that they function as core regulators, integrating multiple physiological processes, including cell growth, tissue formation, and systemic stress responses, throughout the root system.

### 3.3. bZIP Genes in Group III

In Group III, *bZIP* genes show widespread yet finely patterned expression across root tissues, with several members concentrating around developmental hubs. A prominent subset—including *bZIP29*, *bZIP51*, *bZIP28*, *bZIP60*, *bZIP54*, and *bZIP41*—exhibits enriched expression in cells surrounding the quiescent center and meristem zone (Figure 3G–I and Figure S2C). These genes are notably regulated by nitrogen signaling and appear to function in cell fate determination (Figure 3G–I and Figure S2C), tissue patterning, and root cap development. Another expression pattern is observed in transition and maturation zones, where *bZIP18* and *bZIP61* show elevated expression in the epidermis, cortex, endodermis, and stele. Their responsiveness to diverse nutrients and abiotic stresses suggests roles in coordinating cell differentiation with environmental adaptation (Figure 3G–I and Figure S2C). In contrast, *bZIP56* and *bZIP64* demonstrate broad expression spanning all root zones—from the root cap to mature tissues—and respond to UV-B, temperature, and nitrogen signals (Figure 3G–I and Figure S2C). This widespread expression profile indicates these factors may serve as integrative regulators, modulating fundamental physiological processes such as light and temperature adaptation, as well as nutrient response throughout the root system.

### 3.4. bZIP Genes in Group IV

Finally, Group IV *bZIP* genes are organized into clear expression hierarchies that denote functional specialization across root domains. One subset—including *bZIP12*, *bZIP14*, *bZIP36*, *bZIP40*, and *bZIP66*—shows enriched expression in the quiescent center and the surrounding stem cell niche (Figure 3J–L and Figure S2D). These genes are regulated by nitrogen signaling and appear essential for maintaining meristem homeostasis and mediating nutrient-responsive adaptation. In contrast, *bZIP13*, *bZIP35*, and *bZIP39* display low but widespread expression across all root cell types (Figure 3J–L and Figure S2D). Their upregulation under diverse abiotic stresses and in response to nitrogen signaling suggests roles in fine-tuning stress responses or in modulating rare cell populations. Notably, *bZIP37* and *bZIP38* are broadly expressed throughout all root regions and respond to osmotic stress, salt stress, and nitrogen signals (Figure 3J–L and Figure S2D). This expression profile indicates their potential as central coordinators of intercellular communication and systemic stress adaptation across the root system.

## 4. The Molecular Regulatory Mechanism of bZIP Transcription Factors in Root Environmental Adaptation

The bZIP transcription factor in plants plays a crucial role in managing physiological and stress responses at both the transcriptional and post-transcriptional levels. It achieves this by linking its activation to specific intracellular signals via various regulatory mechanisms. These complex processes include promoting the expression and accumulation of bZIPs, processing the precursor mRNA of bZIPs, undergoing reversible post-translational modifications, shuttling between the cytoplasm and nucleus, facilitating long-distance translocation, dimerization, and self-activation. The versatile regulatory mechanisms of bZIPs ensure precise control over developmental processes and environmental adaptation.

### 4.1. Direct Induction of the bZIP Genes’ Expression

**Figure 4:**
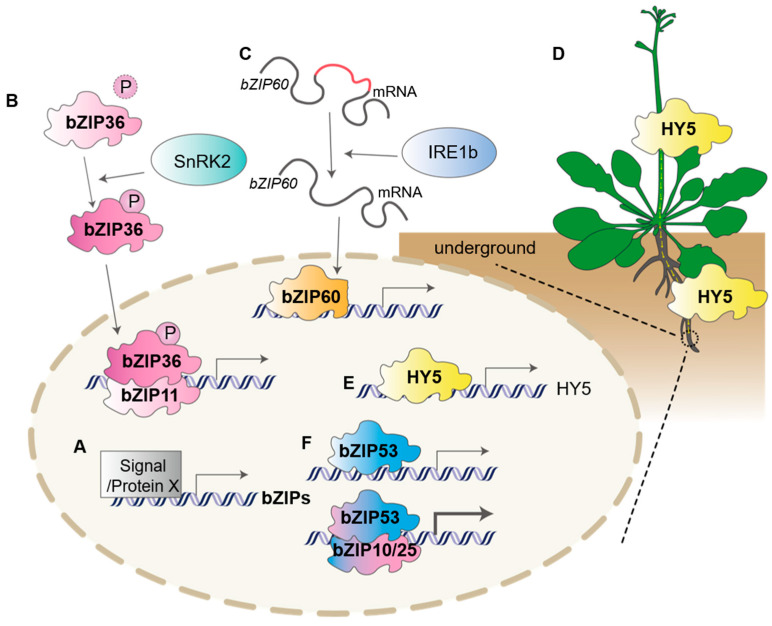
Molecular regulatory mechanisms of bZIP transcription factors in root environmental adaptation. (**A**) Transcriptional regulation: Upstream regulators (X) control bZIP gene expression at the transcriptional level. (**B**) Post-translational modification: SnRK2-mediated phosphorylation promotes nuclear translocation of bZIP36, activating downstream gene; under drought stress, bZIP36-ABI interaction prevents transcriptional activation. (**C**) mRNA processing: ER stress triggers IRE1-mediated splicing of bZIP60 precursor mRNA, generating a truncated protein that enters the nucleus to regulate target genes. (**D**) Long-distance transport: Light signaling promotes shoot-to-root movement of HY5 protein. (**E**) Autoregulation: Shoot-derived HY5 binds G-box elements in its own promoter in root cells, establishing a self-activation loop. (**F**) Dimerization control: bZIP53 heterodimerizes with bZIP10/25 to enhance transcriptional activation of downstream targets.

A variety of environmental signals can directly stimulate *bZIP* genes transcription, making them key players in stress responses (Figure 4A). For instance, under energy- and carbon-starvation conditions, the central kinase KIN10 rapidly induces the expression of *bZIP11* and *bZIP53* genes to initiate metabolic reprogramming [34,35]. Additionally, salt stress promotes metabolic adaptation in the root system by activating parallel bZIP signaling pathways, leading to the expression of multiple members, including *bZIP1* [36]. Furthermore, when zinc is deficient, the expression of *bZIP19* and *bZIP23* is specifically upregulated, activating a series of genes involved in zinc homeostasis to adapt to nutritional stress [37,38]. In rice (*Oryza sativa* L.), under high salt, the key circadian clock protein OsCCA1 can directly bind to the *OsbZIP46* promoter and activate its transcription [39], thereby regulating the expression of downstream ABA-responsive genes. OsABI5, a bZIP family protein in rice, is a key positive regulator of the ABA signaling pathway. The rice PIF family transcription factor OsPIL15 can directly bind to the PBE-box motif (CACATG) in the promoter region of *OsABI5*, thereby activating the transcription of *OsABI5* and regulating the aperture size of stomata [40]. The *bZIP* transcription factor gene can also be directly activated by other bZIP transcription factors. Under low nitrogen stress, OsbZIP79 directly activates *OsABI5* transcription. When melatonin is applied externally, this regulatory module can be enhanced. Subsequently, OsABI5 binds to the promoters of genes involved in reactive oxygen homeostasis and nitrogen metabolism, thereby activating their transcription [41]. Thus, directly inducing *bZIP* gene expression is a common and essential regulatory mechanism for bZIPs participating in environmental adaptation (Figure 4A).

### 4.2. Reversible Post-Translational Modifications

Reversible post-translational modifications, particularly phosphorylation, play a crucial role in precisely regulating the activity, subcellular localization, and protein–protein interaction selectivity of the bZIP protein (Figure 4B). During periods of energy stress, SnRK1 phosphorylates bZIP63, converting it from an inactive homodimer to an active heterodimer and enabling it to interact with proteins such as bZIP11. This modification helps mediate the plant’s response to low-energy conditions [42]. In the context of drought and salt stress, the ABA-mediated pathway is regulated by bZIP transcription factors, such as AREB1/ABFs, which require multi-site phosphorylation by kinases, such as SnRK2, to become activated. This activation is essential for the efficient expression of genes that confer drought-resistance [10,43]. Moreover, phosphorylation also influences the nucleoplasmic transport of the bZIP protein. For example, under mechanical stress, protein phosphatase 2A (PP2A) facilitates the nuclear accumulation of VirE2-interacting protein 1 (VIP1, bZIP51) by dephosphorylating it, which in turn regulates root growth [44]. In the realm of pathogen defense, bZIP10 activity can be modulated by phosphorylation, enabling it to interact with the antagonistic protein Lesions Simulating Disease Resistance 1 (LSD1). This interaction is important for balancing immunity and cell death in response to pathogens [45].

### 4.3. Processing the Precursor mRNA of bZIPs

Plants possess a unique mechanism for processing mRNA that does not depend on traditional spliceosomes. This mechanism plays a crucial role in the rapid activation of membrane-anchored bZIP transcription factors, especially during endoplasmic reticulum (ER) stress responses. When unfolded proteins accumulate in the ER or when cells experience thermal stress, the membrane-bound INOSITOL REQUIRING ENZYME 1 (IRE1) kinase is activated. As a bifunctional enzyme, IRE1 has endonuclease activity that directly recognizes and splices the precursor mRNA of bZIP60. This splicing event removes an intron-like sequence that encodes the C-terminal transmembrane domain, resulting in a frame shift. Ultimately, this process generates a new, active bZIP60 protein that can move freely to the nucleus, thereby initiating the transcription program for downstream stress-related genes [46,47,48] (Figure 4C).

The splicing modification of bZIP mRNA precursors plays a crucial role in plant stress adaptation, as demonstrated by TabZIP74 in wheat—a functional homolog of Arabidopsis bZIP60. This process is part of the unfolded protein response (UPR), which maintains ER homeostasis under stress conditions. In wheat roots, both unspliced (membrane-localized) and spliced (nuclear-localized) isoforms coexist under normal growth, with the spliced form being less abundant [49,50,51]. As a key integrator of abiotic and biotic stress signals, TabZIP74 regulates root development by controlling water absorption capacity, cell proliferation, and lateral root formation. Its mRNA splicing—induced by drought, ABA, and pathogen infection—enables nuclear translocation and activation of downstream targets. Through this splicing-mediated mechanism, TabZIP74 coordinately regulates lateral root development and drought stress responses in wheat, translating environmental cues into adaptive root growth [50].

### 4.4. Cytoplasmic-Nuclear Shuttling

In eukaryotic cells, nuclear-cytoplasmic protein shuttling is the dynamic transport of proteins between the nucleus and the cytoplasm. This process is essential for cells to integrate intracellular signals, respond to the external environment, and maintain homeostasis. Nuclear-cytoplasmic shuttling of bZIP transcription factors enables eukaryotic cells to integrate environmental signals and activate stress responses through distinct proteolytic and mRNA splicing pathways. During ER stress, membrane-anchored bZIP28 is cleaved by S1P/S2P proteases, releasing its N-terminal domain for nuclear translocation, where it forms a complex with NF-Y to activate ER stress-responsive genes [52] (Figure 4C). Alternatively, a faster IRE1-mediated pathway directly splices *bZIP60* mRNA under heat or ER stress, producing a truncated protein that translocates to the nucleus and initiates the unfolded protein response [48] (Figure 4C). Beyond ER stress, this shuttling mechanism also regulates plant immunity. Under normal conditions, bZIP10 is retained in the cytoplasm through interaction with LSD1, which masks its nuclear localization signal. However, upon pathogen infection or oxidative stress, bZIP10 dissociates from LSD1 and translocates to the nucleus to induce defense-related genes, thereby modulating plant immune responses and programmed cell death [45]. Additionally, heat stress induces the translocation of bZIP18 and bZIP52 from the cytoplasm to the nucleus, thereby regulating metabolic pathways and long non-coding RNA (lncRNA) expression [53]. Collectively, these bZIP shuttling mechanisms provide plants with layered regulatory strategies to maintain cellular homeostasis under varying environmental challenges.

### 4.5. Long-Distance Translocation

Recent studies reveal that certain bZIP transcription factors function as mobile long-distance signals, systemically coordinating plant growth across organs. In *Arabidopsis*, the ELONGATED HYPOCOTYL5 (HY5, bZIP56) protein synthesized in shoots is translocated via the phloem to roots, where it enters the nucleus and activates the nitrate transporter gene *NRT2.1* by binding to G-box elements in its promoter. This mechanism directly couples aerial light perception with underground nutrient acquisition, thereby enabling adaptive foraging under low-nitrogen conditions [54] (Figure 4D).

Similarly, TGA7(bZIP50) represents another mobile bZIP protein that balances shoot-root development under nitrogen stress. Grafting experiments confirm its phloem-mediated movement from shoots to roots [55]. In shoots, TGA7 enhances photosynthetic capacity by activating *Lhcb* genes, while in roots, it directly activates *NRT2.1* and indirectly upregulates other nitrate transporters through transcriptional cascades [55]. This dual functionality allows TGA7 to optimize resource allocation by restraining shoot growth while promoting root expansion under nitrogen deficiency [55].

### 4.6. Self-Activation and Dimerization

Self-activation represents an important regulatory property of bZIP transcription factors (Figure 4F), enabling autonomous transcriptional initiation of both target genes and their own expression. Experimental evidence from diverse plant species confirms this capability: in *Eleutherococcus senticosus*, multiple EsbZIP factors (EsbZIP1, EsbZIP4, EsbZIP5) demonstrate autoregulatory activity [56], while in rice, the VIP1 homologs OsbZIP81 and OsbZIP84 exhibit self-activation in both yeast systems and plant cells [57]. This self-activation capacity can be enhanced through dimerization and environmental signals. OsbZIP81 and OsbZIP84 strengthen their transcriptional activity by forming homodimers or heterodimers [57]. Furthermore, external cues such as light regulate self-activation, as demonstrated by *HY5* in *Arabidopsis*, whose root expression is activated by shoot-derived signals and modulated by light conditions [54]. These mechanisms enable bZIP factors to establish self-sustaining regulatory circuits that integrate internal and external signals.

Dimerization serves as a fundamental mechanism underlying the functional diversification of bZIP transcription factors in *Arabidopsis thaliana* (Figure 4E). Through specific pairing mediated by their leucine zipper domains, bZIP proteins form selective heterodimers that significantly enhance their regulatory precision. A key example is bZIP53, which, during seed maturation, preferentially heterodimerizes with bZIP10 or bZIP25 rather than forming homodimers, thereby increasing its affinity for specific cis-elements, such as the proline box, and ensuring accurate activation of storage protein genes [58]. This strategic dimerization not only expands DNA-binding specificity but also creates new regulatory interfaces for integrating diverse cellular signals. Studies have revealed that interaction networks involving multiple bZIP factors can be organized into distinct functional modules, demonstrating how dimerization drives functional specialization [59]. Notably, several bZIP factors, including bZIP11, bZIP44, bZIP53, and bZIP63, employ specific heterodimerization patterns to participate in SnRK1-mediated metabolic processes, ultimately influencing energy distribution throughout the plant, including roots [35,36,42]. Further supporting this mechanism, an example from wheat demonstrates that dimerization also diversifies bZIP functions in cereals. Specifically, the TabZIP11-D heterodimerizes with TabZIP14/36 to regulate cold signaling by transcriptionally activating TaCBF1 [60].

## 5. Physiological Roles of bZIPs in Arabidopsis Root Environmental Adaptation

### 5.1. Plant bZIP Responses to Light Signal

Light signaling regulates root environmental adaptation through bZIP transcription factors via multiple interconnected mechanisms. Specifically, the bZIP factor HY5 functions as a central regulator that can be locally activated in roots through autonomous photoreception and also translocates from the shoot to the root as a long-distance signal [54,61,62,63]. When activated, HY5 in the roots helps maintain a balance of reactive oxygen species (ROS) by directly activating peroxidase genes, such as PER6, while inhibiting their negative regulator, UPBEAT1. This process promotes root meristem activity [63]. Additionally, HY5 integrates light signaling with nutrient signaling by activating the nitrate transporter NRT2.1 and the phosphate response factor LPR1, thereby optimizing nutrient uptake under varying resource conditions [54].

**Figure 5:**
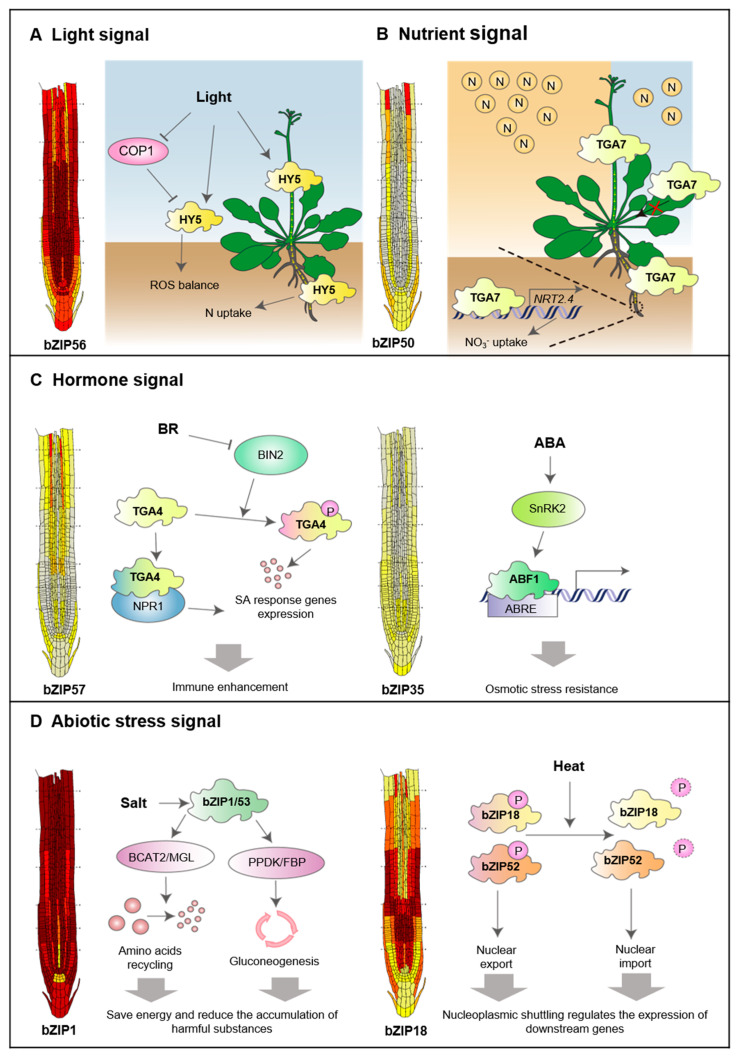
Physiological functions of bZIP transcription factors in *Arabidopsis* root adaptation to the environment. (**A**) Light signal can not only induce the transport of HY5 from the above-ground part to the root cells to promote nitrogen absorption, but also directly activate the *HY5* in the roots to maintain the balance of ROS. (**B**) TGA7 functions as a shoot-to-root mobile bZIP TF in *Arabidopsis* to activate photosynthetic genes in the shoot and nitrate-uptake-related genes in the root to balance shoot and root development. (**C**) BR inhibits BIN2 kinase activity, preventing it from phosphorylating TGA4 and enhancing TGA4-NPR1 binding, thereby strengthening the SA-mediated immune response (**left**). During drought stress, ABA prevents PP2C from binding to SnRK2 kinases, allowing them to phosphorylate downstream AREB/ABF transcription factors and initiate the expression of ABA-responsive genes (**right**). (**D**) bZIPs respond to abiotic stress signals. After salt stress, bZIP1/bZIP53 affects amino acid catabolism and gluconeogenesis regulation, modulating carbon and nitrogen metabolic pathways and enabling cells to conserve energy and reduce the accumulation of harmful substances (**left**). Under heat stress, bZIP18 and bZIP52 undergo dephosphorylation, enabling them to enter the nucleus and regulate downstream gene transcription (**middle**). The presence of bZIP17 and bZIP28 helps cells complete the UPR and ensures normal root development; in their absence, root development is abnormal (**left**).

Under competitive light conditions, perceiving far-red light in shoots leads to an accumulation of HY5 in lateral root primordia. Here, it modulates auxin transport by regulating the levels of PIN3 and LAX3, ultimately inhibiting the emergence of lateral roots [64]. Furthermore, HY5 helps balance carbon and nitrogen by linking carbon fixation in the shoots with nitrate uptake in the roots, creating a feedback loop that maintains metabolic homeostasis [54,61] (Figure 5A). It is important to note that although HY5 mediates communication between shoots and roots, some light responses can occur through pathways that do not involve HY5, via cryptochromes and other downstream signaling components [62]. Overall, these mechanisms enable plants to coordinate root development with surrounding light conditions, thereby enhancing resource acquisition and growth in changing environments.

### 5.2. Plant bZIPs Responses to Nutrient Signals

Nutrient availability—including nitrogen, sugars, and micronutrients—serves as a key environmental cue that directly influences plant growth, metabolic homeostasis, and stress adaptation. bZIP transcription factors act as central regulators in perceiving and transducing these nutrient signals, enabling roots to dynamically adjust their development and physiological functions under fluctuating resource conditions.

bZIPs play an essential role in nitrogen and carbon sensing. In rice, bZIPs are identified through multi-omics analyses as core components of the nitrogen deficiency response network, where they coordinate with other transcription factor families to modulate the expression of N-responsive genes [65]. Similarly, in *Arabidopsis*, bZIP1 functions as a sugar-signaling integrator, rapidly repressing sugar signaling via a hexokinase-dependent pathway and regulating early seedling growth under carbon limitation through heterodimerization with bZIP10 and bZIP63 [66]. Under energy deprivation, bZIP1 and bZIP53 further reprogram amino acid metabolism by forming heterodimers with C-group bZIPs and directly binding to the promoters of key metabolic genes [67,68] (Figure 5D).

Notably, bZIPs also exhibit long-distance and systemic regulation. For example, TGA7 functions as a shoot-to-root mobile transcription factor that coordinates nitrogen-deficiency responses across tissues. It enhances root growth and nitrate uptake by activating photosynthetic genes in shoots and nitrate-uptake-related genes in roots, thereby maintaining shoot–root developmental balance under nutrient stress [55] (Figure 5B right). Systemically, HY5 also moves from shoot to root, synchronizing nitrate uptake with photosynthetic activity to optimize root growth [54] (Figure 4 and Figure 5A). Spatially, in maize, ZmbZIP89 promotes lateral root elongation by activating ZmPRX47 and maintaining ROS homeostasis, thereby improving nutrient acquisition capacity [5].

Additionally, plant roots employ bZIP transcription factors as central regulators to sense and respond to zinc signals. In *Arabidopsis*, bZIP19 and bZIP23 function as zinc sensors by directly binding Zn^2+^ via a conserved zinc-sensor motif, activating the expression of zinc deficiency-responsive genes [69]. These target genes—including multiple ZIP metal transporters such as ZIP4, ZIP3, and ZIP9—are characterized by the presence of specific cis-regulatory palindromic motifs in their promoters [37]. Under zinc-deficient conditions, bZIP19 and bZIP23 form a regulatory module that enhances zinc uptake capacity by inducing ZIP transporter expression in roots [37]. Loss of both bZIP19 and bZIP23 disrupts this adaptive response, leading to hypersensitivity to zinc deficiency and impaired root growth [38,69], demonstrating their essential role in maintaining zinc homeostasis [69] (Figure 5C right).

### 5.3. Plant bZIPs Responses to Diverse Hormone Signals

Plant hormones coordinate growth, development, and environmental responses through intricate interactions with bZIP transcription factors.

Studies have revealed that the brassinosteroid (BR) signaling pathway promotes salicylic acid (SA)-mediated immune responses by inhibiting BIN2 kinase-mediated phosphorylation of the transcription factor TGA3/4 (bZIP22/57), which enhances both TGA3/4-NPR1 interaction and TGA4 protein stability [70,71](Figure 5C left). Phenotypic analyses showed that SA treatment induces primary root elongation, and this SA-induced growth inhibition is further enhanced by BR co-treatment [70]. The spatial expression pattern of TGA4—particularly in the root elongation zone and root cap—correlates with its functional role in integrating SA and BR signals to coordinate pathogen defense.

ABA is a central hormone in plant stress adaptation, primarily enhancing tolerance to osmotic stresses such as drought and salinity. Upon activation by ABA, SnRK2 kinases phosphorylate and activate AREB/ABF transcription factors—including ABF1 (bZIP35), AREB1/ABF2 (bZIP36), ABF3 (bZIP37), and AREB2/ABF4 (bZIP38). These activated factors bind to ABA-responsive elements (ABREs) in the promoters of target genes, thereby driving expression of osmotic-stress-responsive programs [8,72,73] (Figure 5C, right). Further studies indicate that ABF2 is phosphorylated by SnRK2s [73,74], reinforcing their role as master regulators in the SnRK2-ABA signaling cascade. The broad expression of AREB/ABFs across root tissues enables plants to rapidly and precisely initiate adaptive transcriptional responses to water and salt stress.

Auxin serves as the primary hormone regulating root growth, with multiple bZIP transcription factors fine-tuning this process through auxin signaling pathways. For instance, bZIP11, functioning as a low-energy signal-activated regulator, directly activates *IAA3/SHY2* expression in root meristems, suppressing PIN auxin transporters and polar auxin flow to control root apical meristem activity and primary root growth [8]. Similarly, VIP1 translocates to the nucleus under hypo-osmotic and mechanical stress, altering PIN1/PIN2 expression and suppressing root bending by modulating auxin responses [74]. Beyond *Arabidopsis*, the tobacco bZIP factor BZI-1 binds auxin-responsive promoters, such as GH3, fine-tuning auxin-induced transcription and influencing root organogenesis [75]. In poplar, bZIP53 acts as a negative regulator of adventitious root development by directly activating auxin signaling genes *IAA4–1/2*, forming a salt-responsive module that integrates stress signals with auxin pathways to inhibit root growth [76]. These examples collectively demonstrate the conserved role of bZIPs in mediating auxin-regulated root development across species and conditions.

### 5.4. Plant bZIPs Responses to Abiotic Stress Signals

Plant bZIP transcription factors function as central regulators that enable root adaptation to diverse abiotic stresses through integrated molecular networks. These factors coordinate both transcriptional changes and developmental adjustments, optimizing root performance in challenging environmental conditions.

Under drought conditions, bZIP transcription factors activate complementary signaling pathways to enhance stress resilience. The AREB/ABF subfamily acts as master regulators of ABA-dependent responses, binding to ABRE elements and activating stress-responsive genes, such as LEA proteins and PP2Cs, through SnRK2-mediated phosphorylation [77]. In addition to this canonical pathway, bZIPs also regulate root architectural changes. For instance, ZmbZIP4 promotes lateral root formation by activating auxin-related genes, while ZmbZIP89 enhances lateral root elongation by modulating ROS homeostasis via ZmPRX47 [5]. These coordinated mechanisms optimize root system architecture to improve water acquisition while maintaining cellular homeostasis during water deficits.

bZIP factors employ various strategies to address salt stress through both metabolic and structural adaptations. In *Arabidopsis*, bZIP1 and bZIP53 reprogram carbon and nitrogen metabolism by enhancing gluconeogenesis and amino acid catabolism through an ABA-independent SnRK1 signaling pathway [36] (Figure 5D left). Simultaneously, salt-induced ER stress triggers the unfolded protein response (UPR) mediated by bZIP60, which is regulated by zinc homeostasis through the ER transporter ZTP29s [78]. This UPR pathway interacts with redox signaling, establishing a crucial connection between protein folding and oxidative stress management. Developmentally, poplar bZIP53 optimizes root architecture under salinity by negatively regulating adventitious root formation through direct activation of *IAA4* genes [76].

Beyond drought and salinity, bZIP factors mediate adaptation to other environmental challenges. During heat stress, bZIP18 and bZIP52 translocate to the nucleus to regulate metabolic pathways and lncRNA expression [53] (Figure 5D right). Similarly, cold and UV-light responses involve specific bZIP members that fine-tune gene expression to maintain root growth under suboptimal conditions [79,80].

In summary, bZIP transcription factors form a complex regulatory network that translates diverse environmental signals into optimized root development and enhanced stress tolerance, enabling plants to survive and thrive under challenging abiotic stress conditions.

## 6. Conclusions

As one of the most widely distributed and conserved transcription factor families in eukaryotes, bZIPs are key regulators of plant growth, development, and responses to environmental stress. Based on phylogenetic analysis of their three-dimensional domain architectures, plant bZIPs can be classified into four major groups, each performing distinct functions in root development and environmental adaptation (Figure 1, Figure 2 and Figure 3). Members from Groups I to IV participate in nearly all detected environmental signaling pathways in roots, with their expression being tightly regulated by diverse environmental cues.

By integrating bZIP expression data from published *Arabidopsis* root single-cell RNA-seq atlases, we compiled cell-type-specific expression profiles of key *bZIP* members across root tip cell types (Figure 3 and Figure S2). This single-cell-resolution approach revealed previously undetected *bZIPs* by RNA-seq—such as *bZIP23*, *bZIP36*, *bZIP62*, and *bZIP75*—highlighting the power of scRNA-seq to uncover spatially restricted regulatory roles and to shift research focus from tissue-level to cell-specific functional analysis. Moving forward, combining single-cell gene editing, proteomics, and spatial transcriptomics will be essential to dissect the precise functions of bZIPs within specific cell types and to track the dynamic reorganization of bZIP networks in response to environmental changes.

Our integrated analysis further elucidated how representative bZIP members from Groups I–IV engage with multiple signaling pathways through distinct regulatory strategies—including selective dimerization, post-translational modifications, nucleocytoplasmic shuttling, and targeted transcriptional control—to integrate light, hormone, nutrient, and abiotic stress signals (Figure 4 and Figure 5). For example, Group I members such as bZIP1 and bZIP53 modulate carbon–nitrogen balance under salt stress [36]; Group II members bZIP19 and bZIP23 sense zinc availability and activate adaptive gene expression [38,69]; Group III member bZIP29 responds to mechanical stress by regulating cell-cycle and cell-wall genes [6]; and Group IV members bZIP35–bZIP38 mediate ABA-dependent drought responses [73,74]. These layered regulatory mechanisms enable bZIPs to orchestrate transcriptional reprogramming that optimizes root architecture, enhances stress tolerance, and maintains metabolic homeostasis.

In summary, bZIP transcription factors act as pivotal hubs in root environmental adaptation through cell-specific expression and multi-pathway crosstalk. Single-cell technologies have opened new avenues for deciphering their complex functions, and while significant progress has been made in understanding their dynamic regulation, many questions remain. Continued advances in spatial multi-omics and functional genomics will further illuminate how bZIP networks enable plants to adapt to changing environments, offering valuable insights for crop improvement and sustainable agriculture.

## Data Availability

No new data were created or analyzed in this study. Data sharing is not applicable to this article.

## Supplementary Materials

The following supporting information can be downloaded at: https://www.mdpi.com/article/10.3390/ijms27020568/s1.

## Abbreviations

The following abbreviations are used in this manuscript:

bZIP	basic Leucine Zipper
scRNA-seq	single-cell RNA sequencing
uORF2	Up stream Open Reading Frame2
ABA	Abscisic Acid
QC	Quiescent Center
*Os*	*Oryza sativa*
ABI	ABA-responsive
SnRK2	Snf1-related protein kinase 2
ER	Endoplasmic Reticulum
UPR	Unfolded Protein Response
PP2A	Protein Phosphatase 2A
VIP1	VirE2-interacting Protein 1
LSD1	Lesions Simulating Disease Resistance 1
lncRNA	long non-coding RNA
*E**s*	*Eleutherococcus* *senticosus*
IRE1	INOSITOL REQUIRING ENZYME 1
HY5	*Arabidopsis* ELONGATED HYPOCOTYL5
*NRT2.1*	Nitrate Transporter 2.1
ROS	Reactive Oxygen Species
BR	Brassinosteroid
SA	Salicylic Acid
