# Supplementary figures and images for: Plant bZIPs in Root Environmental Adaptation: From Single-Cell Expression Atlas to Functional Insights
Source: Int J Mol Sci. 2026 Jan 6;27(2):568. doi: 10.3390/ijms27020568 (PMC12840723; doi:10.3390/ijms27020568)

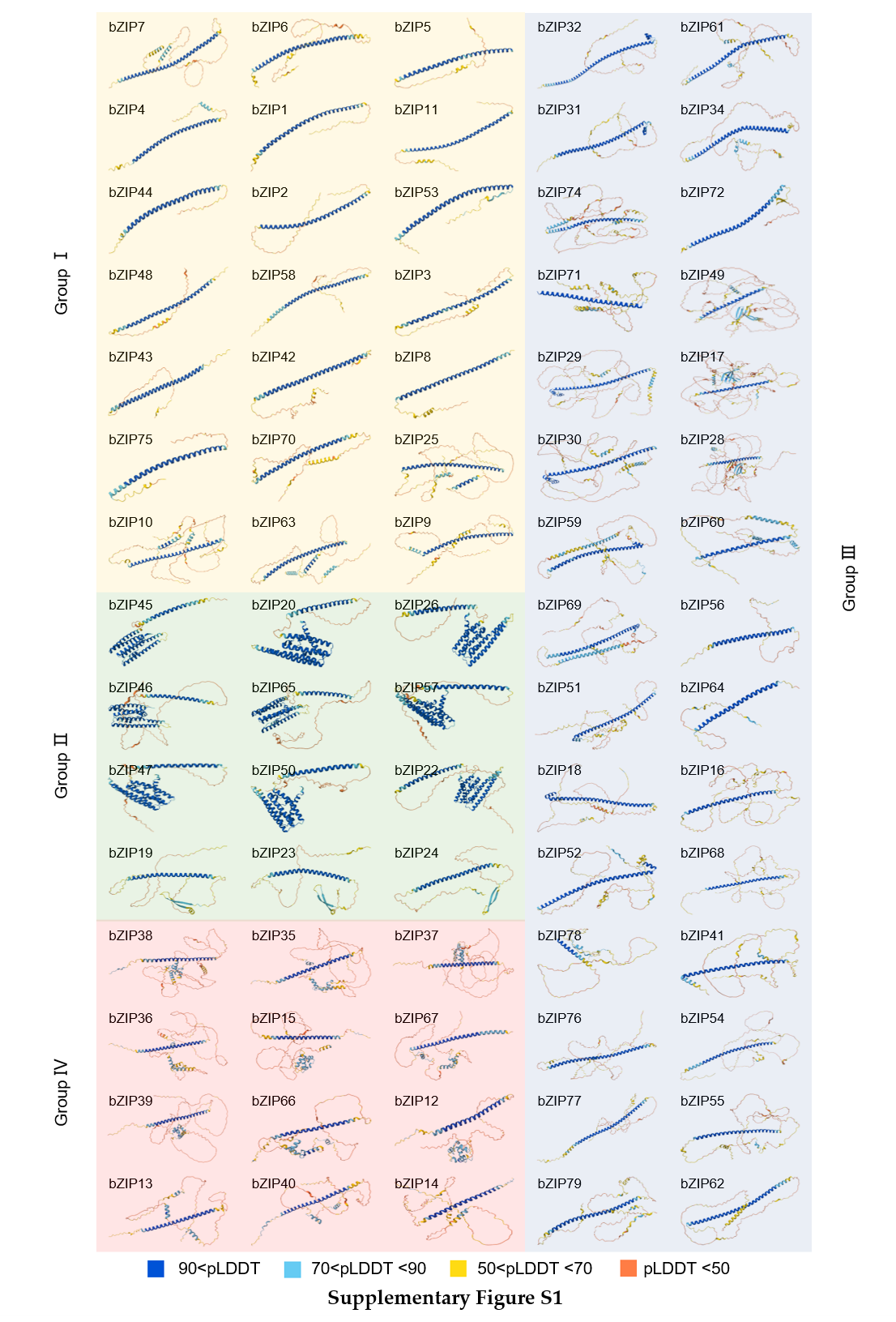

Supplement: Supplementary file 1 [file ijms-27-00568-s001.zip › Supplementary Figures/Supplementary Figures S1.tif]

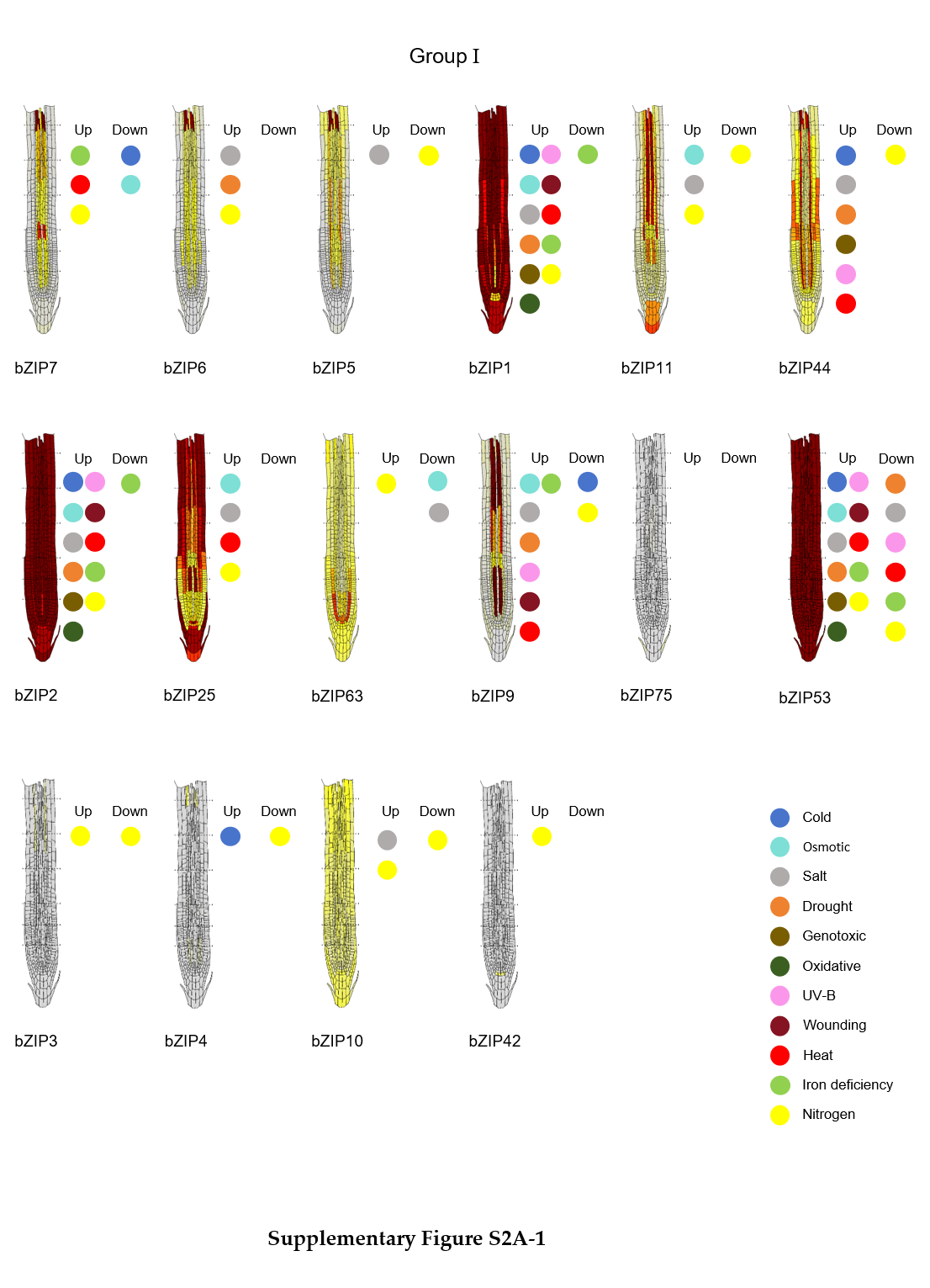

Supplement: Supplementary file 1 [file ijms-27-00568-s001.zip › Supplementary Figures/Supplementary Figures S2A-1.tif]

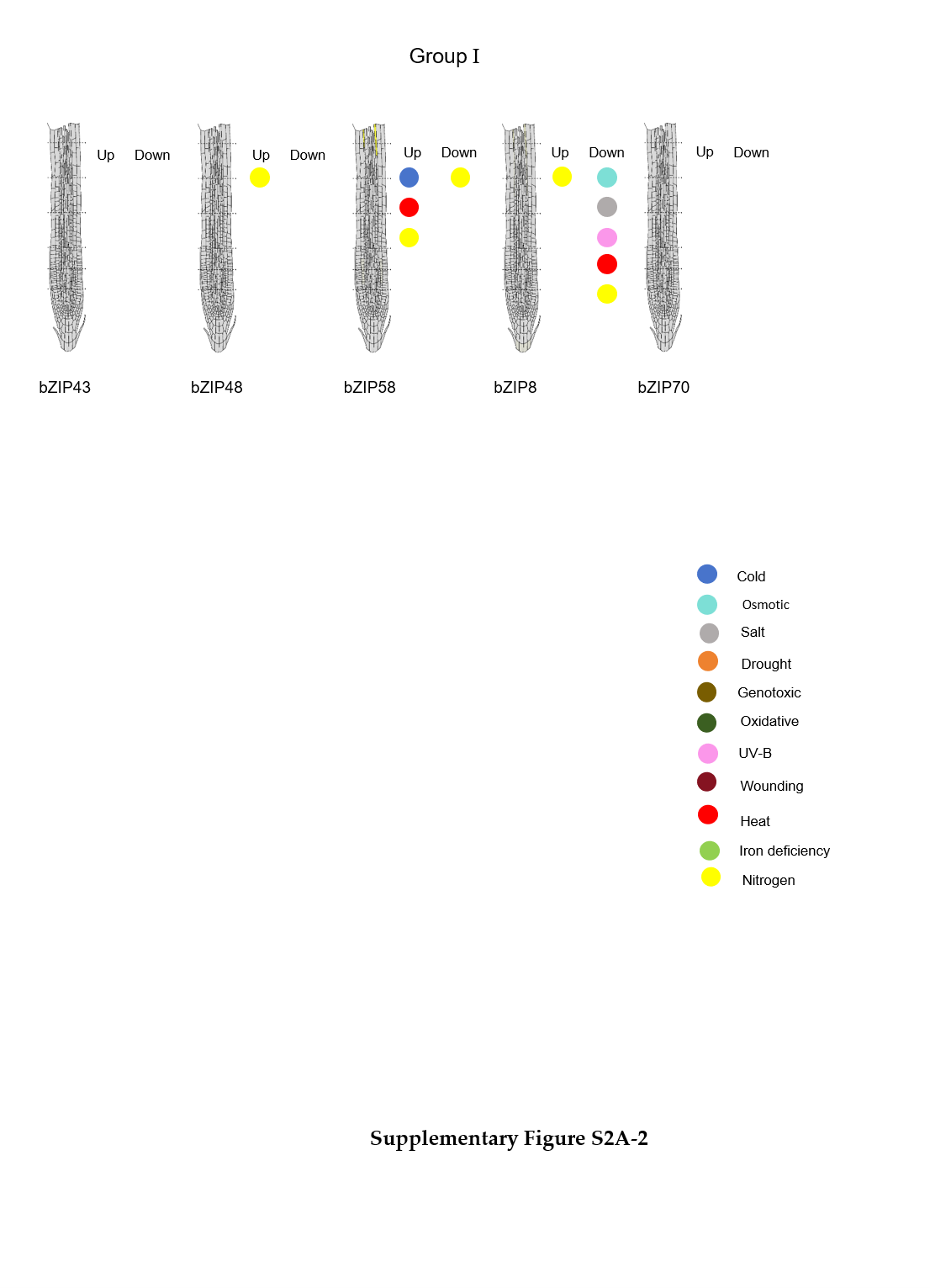

Supplement: Supplementary file 1 [file ijms-27-00568-s001.zip › Supplementary Figures/Supplementary Figures S2A-2.tif]

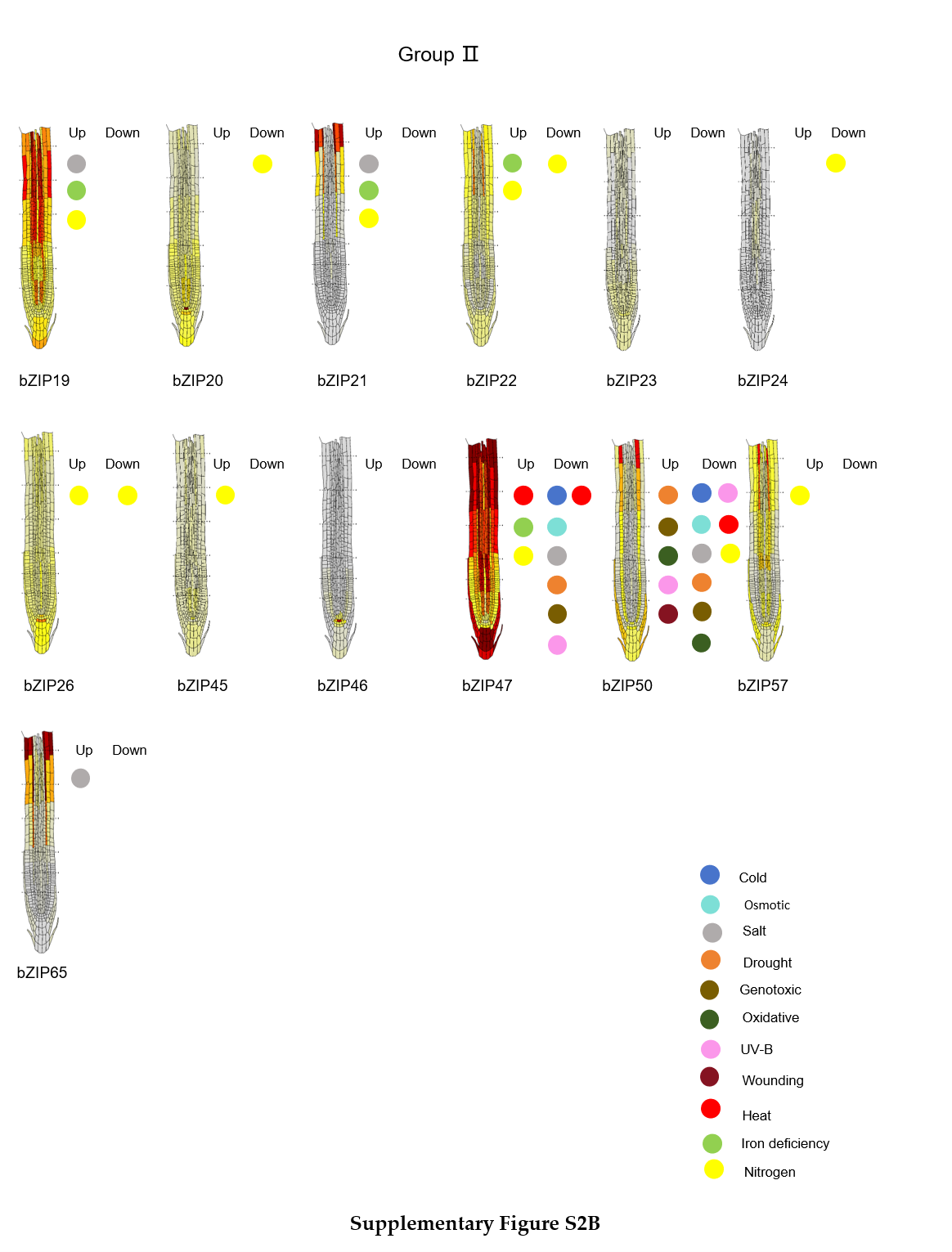

Supplement: Supplementary file 1 [file ijms-27-00568-s001.zip › Supplementary Figures/Supplementary Figures S2B.tif]

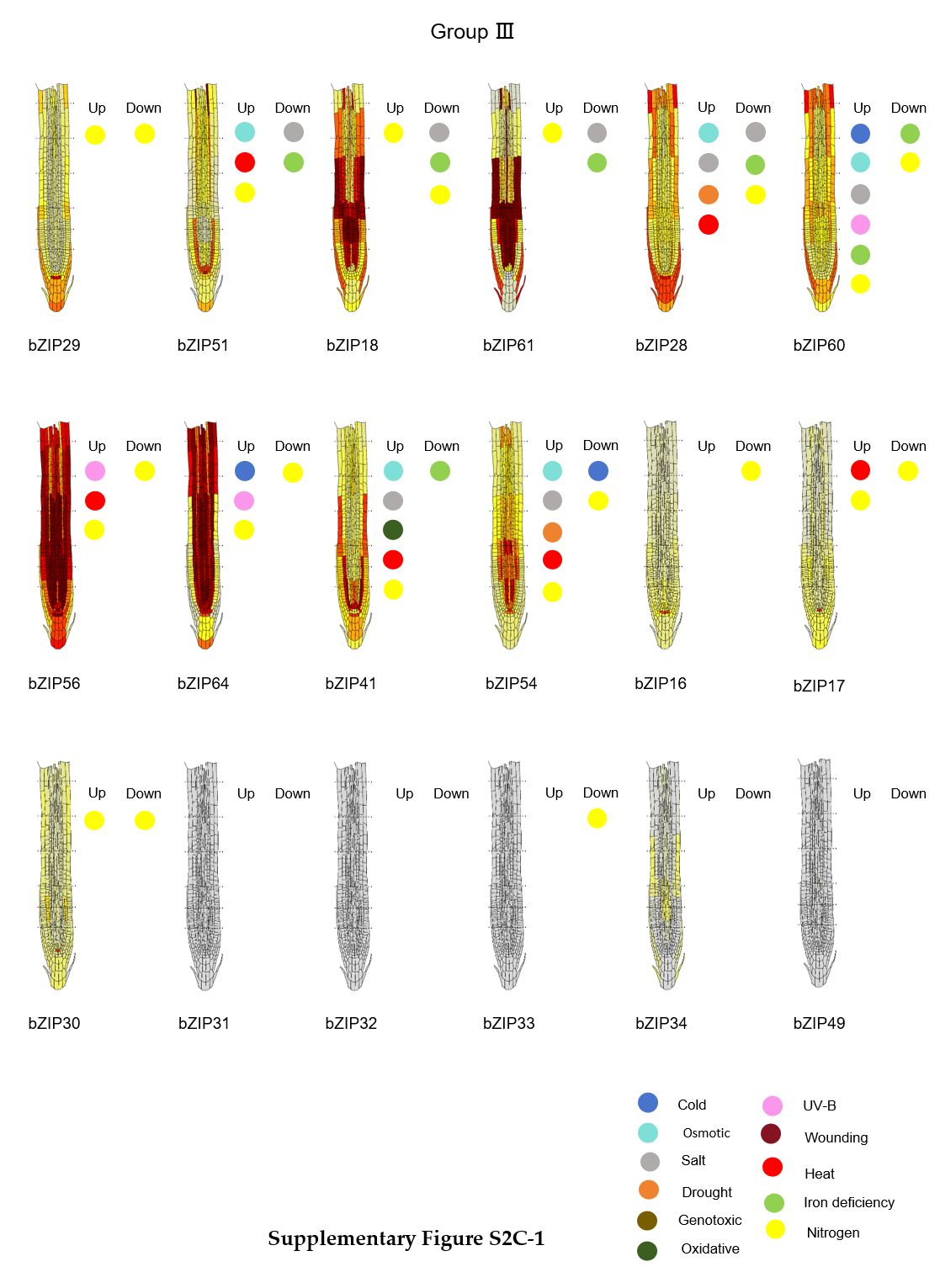

Supplement: Supplementary file 1 [file ijms-27-00568-s001.zip › Supplementary Figures/Supplementary Figures S2C-1.tif]

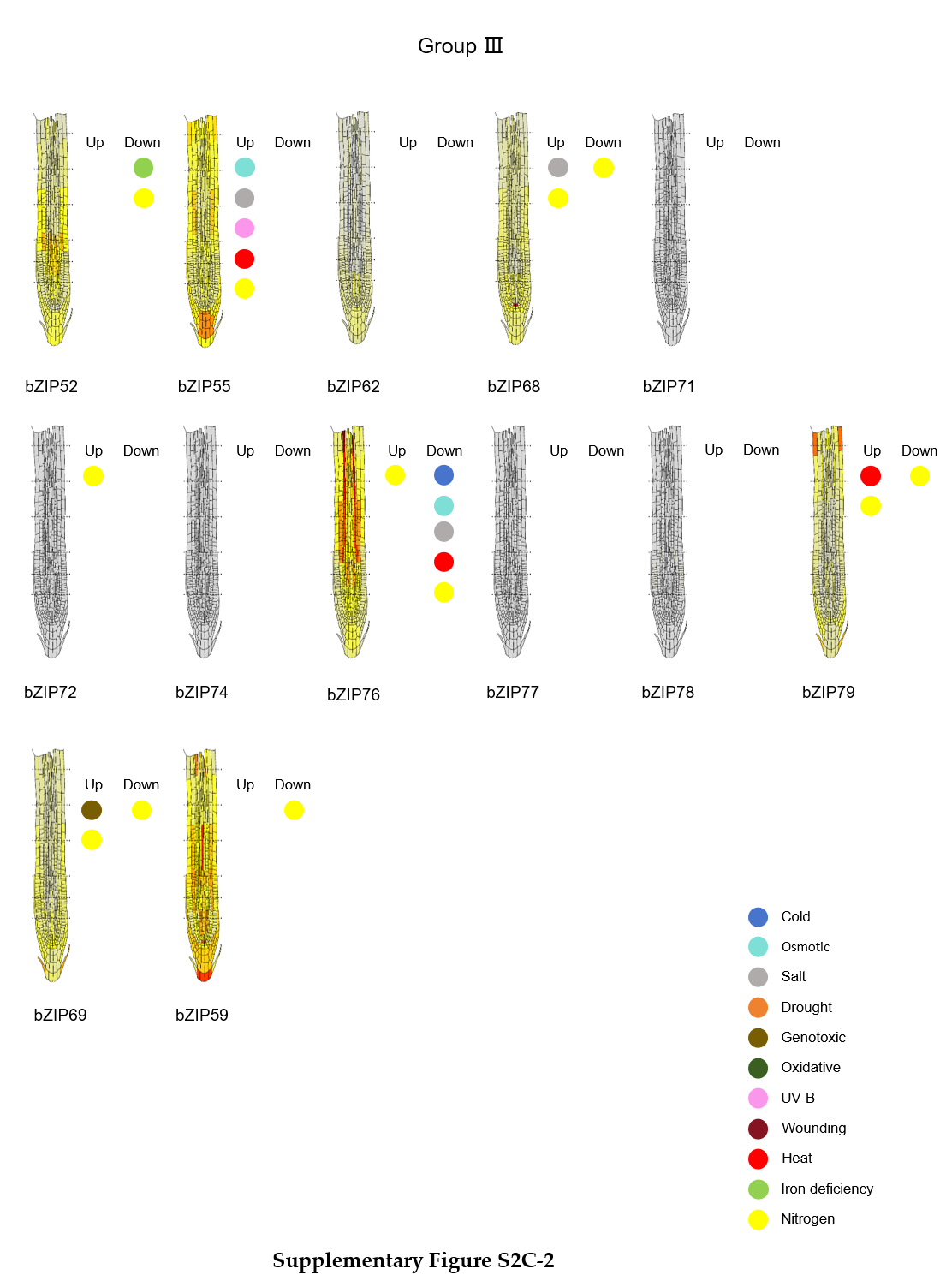

Supplement: Supplementary file 1 [file ijms-27-00568-s001.zip › Supplementary Figures/Supplementary Figures S2C-2.tif]

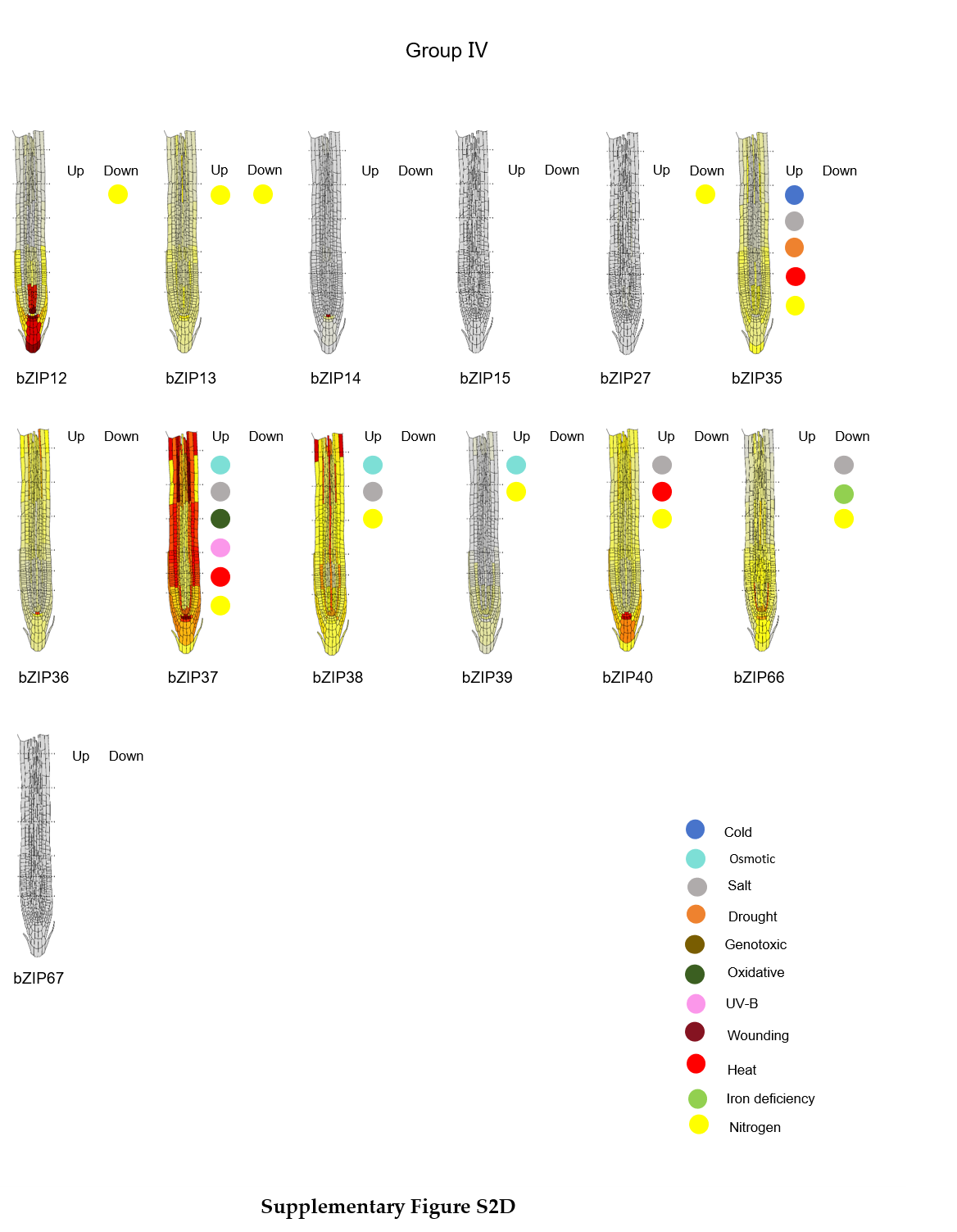

Supplement: Supplementary file 1 [file ijms-27-00568-s001.zip › Supplementary Figures/Supplementary Figures S2D.tif]
